# Cost-Effectiveness of Colorectal Cancer Screening Protocols in Urban Chinese Populations

**DOI:** 10.1371/journal.pone.0109150

**Published:** 2014-10-06

**Authors:** Weidong Huang, Guoxiang Liu, Xin Zhang, Wenqi Fu, Shu Zheng, Qunhong Wu, Chaojie Liu, Yang Liu, Shanrong Cai, Yanqin Huang

**Affiliations:** 1 School of Health Management, Harbin Medical University, Nangang District, Harbin, China; 2 Cancer Institute (Key Laboratory of Cancer Prevention and Intervention, Ministry of Education of China; Key Laboratory of Molecular Biology in Medical Sciences, Zhejiang Province, China), The Second Affiliated Hospital, Zhejiang University School of Medicine, Hangzhou, China; 3 School of Public Health, La Trobe University, Melbourne, Victoria, Australia; University Hospital Heidelberg, Germany

## Abstract

Colorectal cancer (CRC) takes a second and fourth position in the incidence and mortality lists respectively among all malignant tumors in urban populations in China. This study was designed to evaluate the cost-effectiveness of two different CRC screening protocols: faecal occult blood test (FOBT) alone, and FOBT plus a high-risk factor questionnaire (HRFQ) as the respective initial screens, followed by colonoscopy. We developed a Markov model to simulate the progression of a cohort of 100,000 average risk asymptomatic individuals moving through a defined series of states between the ages of 40 to 74 years. The parameters used for the modeling came from the CESP (Comparison and Evaluation of Screening Programs for Colorectal Cancer in Urban Communities in China) study and published literature. Eight CRC screening scenarios were tested in the Markov model. The cost-effectiveness of CRC screening under each scenario was measured by an incremental cost-effectiveness ratio (ICER) compared with a scenario without CRC screening. The study revealed that a combined use of FOBT and HRFQ is preferable in CRC screening programs as an initial screening instrument. Annual FOBT+HRFQ screening is recommended for those who have a negative initial result and those who have a positive result but have failed to continue to colonoscopic examination. Repeated colonoscopy (for those with a positive result in initial screening but a negative colonoscopy result) should be performed at a ten-year interval instead of one-year. Such a protocol would cost 7732 Yuan per life year saved, which is the most cost-effective option. In conclusion, the current Chinese Trial Version for CRC Screening Strategy should be revised in line with the most cost-effective protocol identified in this study.

## Introduction

Colorectal cancer (CRC) is one of the most prevalent cancers in the world [1]. With high levels of incidence and mortality, CRC imposes a significant and potentially avoidable public health burden in most industrialized countries [2], including the United States, Australia and European countries [3]–[5]. In China, CRC has attracted increasing attention over recent years, taking a second and fourth position in the incidence and mortality lists respectively among all malignant tumors in urban populations [6]. The National Plan for Cancer Prevention and Control in China (2004–2010) identified CRC as one of the highest priorities for intervention [7].

CRC is characterized by high prevalence, a long asymptomatic period and eminently treatable precancerous lesions, which together suggests that screening is a prudent option. It has been reported in the literature that CRC screening can reduce mortality effectively and even curb incidence as a consequence of polyp removal [8].

There are several protocols already in existence regarding population CRC screening: the most common interventions being Faecal Occult Blood Test (FOBT), flexible sigmoidoscopy, and colonoscopy. The effectiveness of FOBT has been established by randomized clinical trials [9], and population-based screening using FOBT can reduce mortality by one third [10], [11]. The European Community and United State Multi-Society Task Force on CRC recommend an annual FOBT as one of multiple options for screening individuals at average risk of CRC [12], [13]. The Asia Pacific Working Group Consensus Guideline (APWGCG) recommends FOBT as the first choice for CRC screening in resource-limited countries [14]. However, using FOBT alone as a screening instrument may fail to detect lesions due to intermittent bleeding from CRC and precancerous polyps or in circumstances where small colorectal neoplasia have little or no tendency for bleeding.

Based on a series of CRC screening efficacy studies [15]–[17], the Ministry of Health of China proposed a two-step protocol for population-based CRC screening: (1) an initial FOBT and high-risk factor questionnaire (HRFQ) followed by (2) a full colonoscopy for those suspected cases identified from the initial screening [18]. Arguably, the choice of CRC screening protocols in resource limited settings should be predicated upon evidence of cost-effectiveness considering a wide range of factors such as sensitivity, specificity, acceptability, feasibility, affordability, compliance, and clinical capacity. Many countries such as the USA, Australia, Europe and some Asian countries have sought economic evaluation of their chosen screening protocols for CRC [19]. To our knowledge no such study in mainland China has yet been reported to date (despite extensive enquiry).

In this study, we evaluated the cost-effectiveness of two different CRC initial screening strategies (FOBT vs FOBT+HRFQ) using the Markov model.

## Materials and Methods

### Ethics Statement

This study was approved by the Institutional Review Board of Clinical Research, the Second Affiliated Hospital, Zhejiang University School of Medicine, and was completed in accordance with the ethical principles of the declaration of Helsinki. Written informed consent was sought and obtained from participants prior to the study.

### Study Design

Data for this study came from the project “Comparison and Evaluation of Screening Programs for Colorectal Cancer in Urban Communities in China” (CESP) and published literature. The CESP project was undertaken from July 2006 to December 2008. A total of 400,000 urban residents aged from 40 to 74 years in Hangzhou, Shanghai and Harbin were approached by their local CDC (Center for Disease Control and Prevention) officials, who explained the study to them in detail. Those who agreed to participate in the study were asked to take a FOBT test and fill in a HRFQ. Individuals having one or more of the following features were identified as “risk positive” by the HRFQ: (1) first-degree relative(s) with CRC; (2) a personal history of cancers or intestinal polyps; (3) two or more of the symptoms/histories: (3a) chronic diarrhea; (3b) chronic constipation; (3c) mucous and bloody stool; (3d) history of appendicitis or appendectomy; (3e) history of chronic cholecystitis or cholecystectomy; (3f) history of psychological trauma (e.g. divorce, death of relatives). The participants with either a positive FOBT or a positive HRFQ were offered colonoscopic examination. Any polyps detected during the colonoscopy were removed immediately and sent for histological diagnosis by a pathologist. Those participants who had polyps removed were initially counselled and then followed up three years later with another colonoscopy. The “positive” participants without detected polyps had a second FOBT and HRFQ one year after the initial colonoscopy. Participants with a negative FOBT and those who did not undertake a FOBT screening or colonoscopy were monitored through a routine cancer registry system. Cancers diagnosed by medical facilities are reported to the cancer registry system.

### CRC screening protocols tested in this study

We compared two initial screening protocols: (1) FOBT alone and (2) FOBT plus HRFQ. In both protocols, individuals who were considered of interest were offered a colonoscopic examination.

1. FOBT as an initial screening instrument

Four scenarios were developed for protocol one (Figure S1).

**Scenario A_1_**: The participants take a FOBT. Those with a FOBT positive result are offered a colonoscopy. Polyps (if found) are removed during the colonoscopic examination and follow-up colonoscopy is undertaken every three years for those with polyps removed. Those participants without polyps are offered another FOBT in ten years. Participants with an initial negative FOBT result or those having an initial positive FOBT but for whatever reasons elect not to comply with the recommended procedures were offered an annual follow-up FOBT.

**Scenario A_2_**: Similar to *Scenario A_1_*; however, those participants with an initial negative FOBT result or those having a positive FOBT initially but for whatever reasons elect not to comply with the recommended procedures were monitored through a routine cancer registry system.

**Scenario A_3_**: Similar to *Scenario A_1_*; however, those participants without polyps take part in an annual follow-up colonoscopy instead of a 10 year interval.

**Scenario A_4_**: Similar to *Scenario A_3_*; the only difference is that the participants with an initial negative FOBT result or those having a positive FOBT initially but for whatever reasons elect not to comply with the recommended procedures were monitored through a routine cancer registry system.

1. FOBT plus HRFQ as an initial screening instrument

Four scenarios were developed for protocol two (Figure S2).

**Scenario B_1_** Participants are offered a FOBT and a HRFQ. Those resulting in a positive outcome (either FOBT or HRFQ) are offered a colonoscopic examination. The follow-up procedures are similar to those of *Scenario A_1_*.

**Scenario B_2_** Similar to *Scenario B_1_*; however, participants with an initial negative result(both FOBT and HRFQ)or those with a positive initial result but for whatever reasons elect not to comply with the recommended procedures were monitored through a routine cancer registry system.

**Scenario B_3_** Similar to *Scenario B_1_*; however, participants without polyps are offered an annual follow-up colonoscopy instead of a 10 years interval.

**Scenario B_4_** This is the scenario currently implemented in China. Participants are offered a FOBT and a HRFQ. Those with a positive result (either FOBT or HRFQ) are offered a colonoscopic examination. Polyps (if present) are removed during the colonoscopic examination and follow-up colonoscopy is undertaken every three years. Those without polyps take part in annual follow-up FOBT and HRFQ. The participants with a negative result(both FOBT and HRFQ)initially and those having a positive result initially but for whatever reasons elect not to comply with the recommended procedures were monitored through a routine cancer registry system.

### Markov Model

We estimated costs and effectiveness of these eight scenarios using the Markov model, a transitional probability model. The Markov model allows us to simulate the trajectory of a hypothetical cohort through different health states [20]. A Markov model describes the probabilities of particular transitions of a particular group of people from one health state to another over a defined period of time. The health states are divided into transient states and absorbing states. A transient state can change to another transient state or to an absorbing state; whereas an absorbing state (such as death) cannot change to other states (such as normal, polyp, CRC) [20]. We developed the Markov model using Microsoft Excel to simulate the progression of a cohort of 100,000 average-risk asymptomatic individuals moving through a defined series of states from 40 to 74 years. In this simulation, the health states of individuals were categorized either as normal, polyp, CRC or death. After successive iterations, the model estimated the cumulative costs and effectiveness for the entire cohort over a35 year period. Each resultant simulation was compared with that of a scenario in which no screening is involved.

### Transitional Parameters

The simulation model was developed using Chinese population data. Some transitional parameters were borrowed from studies in other countries if they were not available in China.

The CESP project provided most of the clinical, epidemiological and costing data. It revealed that 45.37% and 53.22% eligible participants complied with the initial FOBT and FOBT+HRFQ requests respectively. Some 37.32% FOBT positive participants and 46.78% FOBT+HRFQ positive participants accepted the offer of colonoscopy. Every participant with polyps had polypectomy, amongst whom 32.07% resulted from FOBT screening alone, and 26.13% from FOBT+HRFQ screening. Previous studies showed that colonoscopic polypectomy can probably reduce CRC incidence by around 76–90% [21]. For this study, we assumed a conservative reduction of 75% CRC incidence following colonoscopic polypectomy. The sensitivity and specificity of FOBT were found to be 42.90% and 86.10%, respectively. The sensitivity of FOBT+HRFQ increased to 88.90%, while its specificity decreased to 71.70% [16], [22]. The incidence and fatality data used in the simulation model came from the Chinese Cancer Registry Annual Reports [6] and the 5th National Census [23] (Table 1).

**Table 1:** Parameters used for the modeling of CRC screening protocols.

Variable	Values (range)	Ref.
Sensitivity of FOBT	42.90% (20%–60%)	[16], [22]
Sensitivity of FOBT+HRFQ	88.90% (75%–90%)	[16], [22]
Specificity of FOBT	86.10% (50%–90%)	[16], [22]
Specificity of FOBT+HRFQ	71.70% (50%–90%)	[16], [22]
Coverage of FOBT	45.37% (30%–100%)	CESP
Compliance with colonoscopy request after initial screening by FOBT	37.32% (30%–100%)	CESP
Coverage of FOBT plus HRFQ	53.22% (30%–100%)	CESP
Compliance with colonoscopy request after initial screening by FOBT+HRFQ	46.78% (30%–100%)	CESP
Polypectomy in people screened by FOBT	32.07%	CESP
Polypectomy in people screened by FOBT+HRFQ	26.13%	CESP
CRC prevented by colonoscopy	75%	[21]
Discount rate	3% (0%–7%)	[24]
Cost (Yuan)		
Marketing for FOBT	1	CESP
Marketing for FOBT+HRFQ	1	CESP
Material of FOBT	5	CESP
Material of FOBT+HRFQ	7	CESP
Distribution and return of FOBT	3	CESP
Distribution and return of FOBT+HRFQ	3	CESP
Pathology	150	BNHI
Colonoscopy	290	BNHI
Polypectomy	500	BNHI
Treatment of CRC	41602	BNHI

Note: CRC - Colorectal Cancer; FOBT - Faecal Occult Blood Test; HRFQ - High-Risk Factor Questionnaire; CESP - Comparison and Evaluation of Screening Programs for Colorectal Cancer in Urban Communities in China; BNHI – Bureau of National Health Insurance.

### Cost estimates

Only direct costs were estimated in this study by the third-party payer's perspective, which included costs associated with initial screening, colonoscopy, polypectomy, pathology tests, and treatment of CRC. The initial screening costs comprised expenses in marketing, materials and reagents for FOBT and HRFQ, and distribution and return of FOBT and HRFQ. These were calculated using the CESP data. All other costs were calculated based on the claim data of the Bureau of National Health Insurance (BNHI). All costs are expressed in Chinese Yuan in this paper and are inflated to the 2008 price level.

### Effectiveness of CRC screening

The effectiveness of CRC screening was presented in terms of “Life Years” saved by the screening. It was calculated through estimating premature deaths (from 40 to 74 years old) as a result of CRC using an age-dependent formula for each age group. The “Life Years” saved under each screening scenario equals to the difference in life years lost between the tested screening scenario and the scenario without any screening. In this study, discount rates for both future costs and future life years were set at 3% [24].

### Cost-effectiveness indicator

We used an Incremental cost-effectiveness ratio (ICER) to measure the cost-effectiveness of the tested screening protocols, defined as the “difference in costs divided by the corresponding difference in effectiveness”. A smaller ICER indicates lower cost for saving one life year, reflecting improved cost-effectiveness.

### Sensitivity analysis

In the sensitivity analysis, we tested the impact of several parameters such as compliance, sensitivity, specificity, and discount rate on the robustness of the simulation model. One-way and two-way sensitivity analyses were applied to assess the influence of those parameters on ICER. The ranges of parameter variations were set as: FOBT - 30% to 100% for compliance; 20% to 60% for sensitivity; and 50% to 90% for specificity; FOBT plus HRFQ - 30% to 100% for compliance; 75% to 90% for sensitivity; and 50% to 90% for specificity; Colonoscopy - 30% to 100% for compliance; Discount rate - 0% to 7% (Table 1).

## Results

### Costs

When no screening was performed, the accumulated expenses over 35 years were estimated through 35 successive iterations in Markov modeling, which resulted in a total of 44,733,623 Yuan for 100,000 average-risk asymptomatic individuals aged 40 years. The total costs under the screening scenario would increase compared to that without screening, with *Scenario A_2_* having the lowest and *Scenario B_3_* having the highest costs. Nevertheless, CRC treatment costs were lower under all screening scenarios compared with those without screening (Table 2).

**Table 2:** Outcome of simulated Markov model for cost-effectiveness of CRC screening.

	*No Screening*	*Scenario A_1_*	*Scenario A_2_*	*Scenario A_3_*	*Scenario A_4_*	*Scenario B_1_*	*Scenario B_2_*	*Scenario B_3_*	*Scenario B_4_*
**Direct cost**									
Marketing	0	790409	44570	889328	44828	777263	53234	1025147	53947
Distribution and return of FOBT or FOBT+HRFQ	0	2371227	222852	2667985	224141	2371227	159703	3075442	161842
Material of FOBT	0	3952045	133711	4446641	134485	0	0	0	0
Material of FOBT+HRFQ	0	0	0	0	0	5440842	372640	7176032	377631
Pathology	0	2201185	124852	2476131	125575	3362131	231199	4432781	234295
Colonoscopy	0	13269796	752667	14927305	757024	24876083	1710618	32797719	1733530
Polypectomy	0	7337282	416173	8253770	418582	11207104	770663	14775938	780985
Treatment of CRC	44733623	35998807	44343703	39542239	44676347	26796779	44103714	32732661	44502127
Total	44733623	65920750	46038528	73203398	46380983	74791992	47401771	96015722	47844357
△Cost	0	21187127	1304905	28469775	1647359	30058369	2668148	51282098	3110734
**Effectiveness**									
Discounted life years lost, Yr	9918	8033	9847	8765	9890	6030	9754	7251	9851
Life years saved, Yr	0	1885	71	1153	28	3888	164	2667	67
Life years saved, %	0	19.01	0.71	11.63	0.28	39.20	1.66	26.89	0.68
CRC accumulated cases, N	2131	1710	2123	1884	2129	1269	2115	1560	2127
CRC deaths, N	1984	1593	1977	1754	1983	1182	1969	1452	1981
CRC prevented, N	0	421	7	247	1	862	16	571	3
CRC prevented, %	0	19.74	0.35	11.60	0.07	40.47	0.75	26.80	0.16
△Effectiveness	0	1885	71	1153	28	3888	164	2667	67
**ICER**	0	11236	18404	24689	59272	7732	16223	19227	46347

Note: CRC - Colorectal Cancer; FOBT - Faecal Occult Blood Test; HRFQ - High-Risk Factor Questionnaire; ICER - Incremental Cost-Effectiveness Ratio.

### Effectiveness

The simulation identified 2131 cases of CRC when no screening was adopted, representing a loss of 9918 CRC-related discounted life years: screening prevents CRC and reduces the loss of CRC-related life years. The highest level of effectiveness was achieved under *Scenario B_1_*, which reduced 40.47% (862 cases) of CRC and avoided 39.20% of loss of CRC-related life years (3888 discounted life years) compared with those without screening (Table 2).

### Costs-effectiveness

For every life year saved,7732 Yuan would be needed under *Scenario B_1_*, 11,236 Yuan under *Scenario A_1_*, 18,404 Yuan under *Scenario A_2_*, 24,689 Yuan under *Scenario A_3_*, 59,272 Yuan under *Scenario A_4_*, 16,223 Yuan under *Scenario B_2_*,19,227 Yuan under *Scenario B_3_*, and 46,347 Yuan under *Scenario B_4_*.*Scenario B_1_* is the most cost-effective protocol among all the scenarios.

### Sensitivity analysis

A greater change in ICER was found when colonoscopy request compliance increased compared with that when coverage of initial screening increased. Colonoscopy compliance also mediated the impact of initial screening coverage on ICER. ICER was more sensitive to changes in initial screening coverage when colonoscopy compliance was higher (Table 3).

**Table 3:** Impact of compliance of initial screening and colonoscopy request on ICER: two-way sensitivity analysis.

Compliance with Colonoscopy	Range of coverage of initial screening	*Scenario A_1_*	*Scenario A_2_*	*Scenario A_3_*	*Scenario A_4_*	*Scenario B_1_*	*Scenario B_2_*	*Scenario B_3_*	*Scenario B_4_*
30%	30%–100%	10819–10558	18794–18748	21654–21696	52882–52890	7504–7156	16238–16290	16843–16893	41307–41320
50%	30%–100%	11984–11247	17324–17501	29289–29395	68978–68923	8636–7916	15510–16044	24319–24449	57240–57138
70%	30%–100%	12599–11309	15396–15799	35276–35476	81265–80960	9071–8370	14177–15253	30271–30511	69725–69141
100%	30%–100%	13035–10883	12846–13562	42065–42467	93806–92387	9105–9518	12208–14167	37171–37631	83269–80430

Note: ICER - Incremental Cost-Effectiveness Ratio.

**Figure 1:**
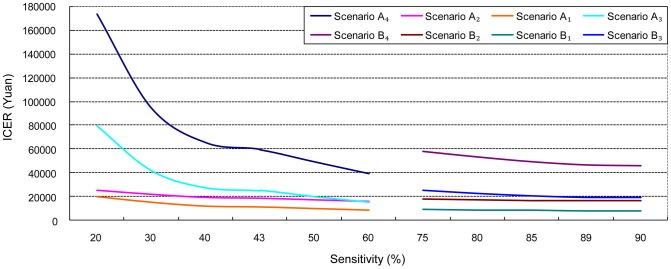
ICER decreases with rising sensitivity of FOBT or FOBT+HRFQ: One-way sensitivity analysis.

ICER decreased with rising sensitivity of initial screening. *Scenario A_3_* and *A_4_* were more sensitive to changes in FOBT sensitivity than *Scenario A_1_* and *A_2_*. When FOBT sensitivity surpassed 42.9% (the parameter used in the modeling), changes in ICER had slowed down dramatically. Moderate changes in ICER were found when the sensitivity of FOBT+HRFQ increased (Figure 1).

**Figure 2:**
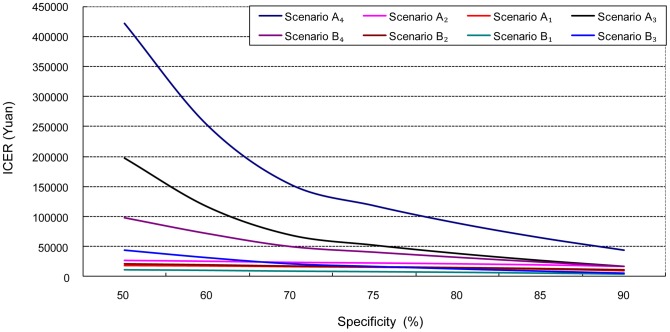
ICER decreases with rising specificity of FOBT or FOBT+HRFQ: One-way sensitivity analysis.

Similarly, ICER decreased with rising specificity of initial screening. *Scenario A_3_* and *A_4_* were more sensitive to changes in specificity of initial screening than other scenarios. When the specificity of initial screening surpassed 86.1% for FOBT or 71.7% for FOBT+HRFQ (the parameters used in the modeling), changes in ICER had slowed down dramatically (Figure 2).

**Figure 3:**
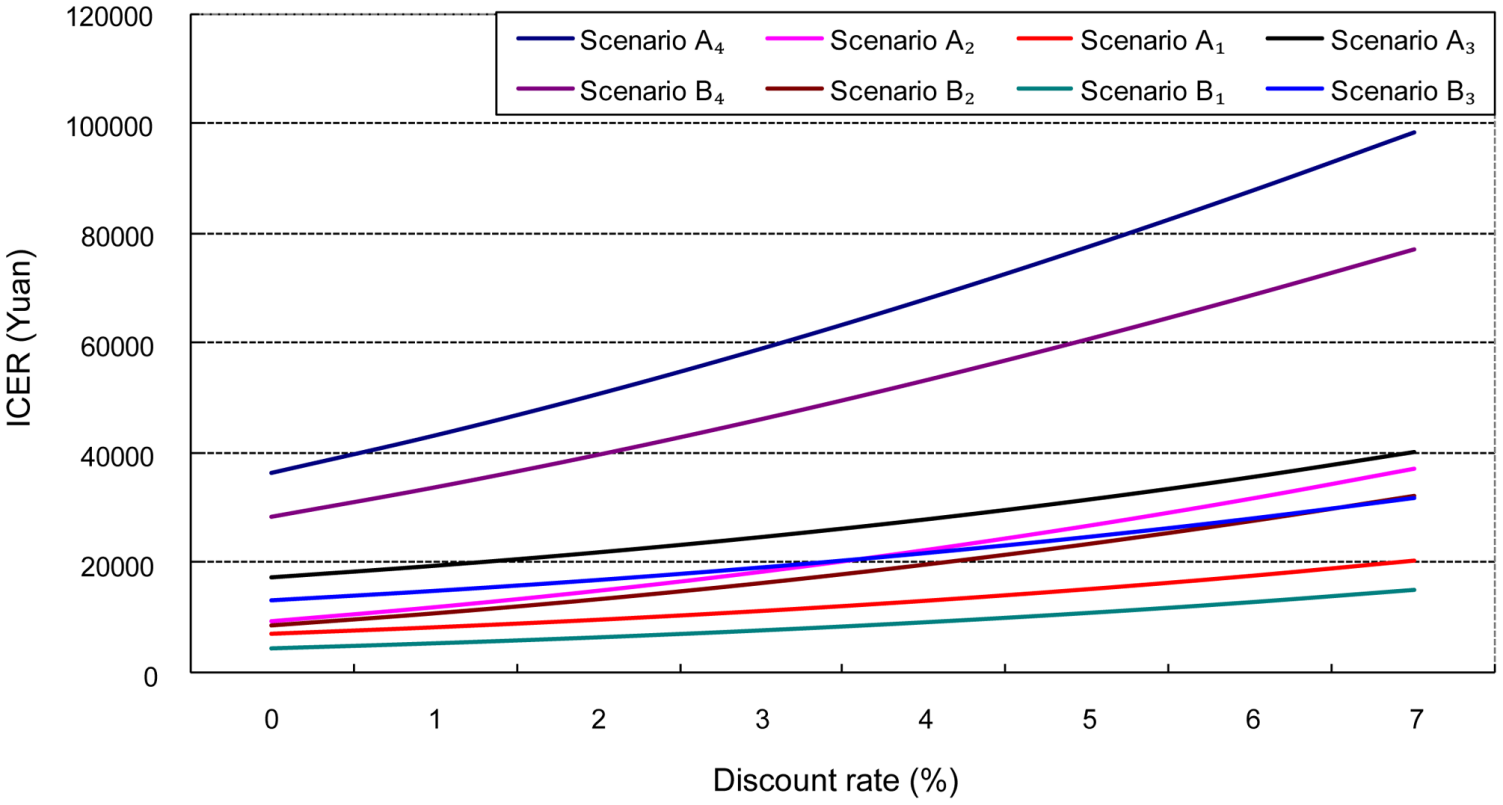
ICER increases with discount rate: One-way sensitivity analysis.

ICER increased with rising discount rate. The ranking order of the eight scenarios in ICER remained largely unchanged with the increase of discount rate, except for *Scenario B_3_*. *Scenario B_3_*was less sensitive to rising discount rate than the others (Figure 3).

*Scenario B_1_* proved to be the most cost-effective, regardless of how the above mentioned parameters changed.

## Discussion

The Markov model simulation revealed that *Scenario B_1_* is the most cost-effective protocol for CRC screening, followed by *Scenario A_1_*, *B_2_*, *A_2_*, *B_3_*, *A_3_*, *B_4_*and *A_4_*. The cost per life year saved under *Scenario B_1_* is the lowest, regardless how simulation parameters were set or changed.

This finding indicates that a combined use of FOBT and HRFQ as an initial step for CRC screening is a better strategy than FOBT alone. Although this means an increase of costs, a greater level of effectiveness can be achieved. This study demonstrated that the costs of protocol two (FOBT+HRFQ as initial screening) under different scenarios are consistently higher than those of protocol one (FOBT as initial screening) under corresponding scenarios (i.e. *A_1_vsB_1_*, *A_2_vsB_2_*, *A_3_vsB_3_*, *A_4_vsB_4_*). However, the effectiveness of protocol two is consistently better than that of protocol one. In addition, regardless how simulation parameters were set or changed, the ICER of protocol two are always lower than those of protocol one.

For people having an initial negative screening result and those having a positive result but failing to comply with the recommended procedures, repeating the initial screening annually can produce a more cost-effective result than routine cancer registry only. This study showed that, for both protocol one and protocol two, scenarios with a repeated initial screening incurred greater costs consistently compared with their alternative counterparts requesting routine cancer registry only (*A_1_ vs A_2_*; *A_3_ vs A_4_*; *B_1_ vs B_2_*; *B_3_ vs B_4_*). However, the effectiveness and cost-effectiveness as measured by ICER of those scenarios with a repeated initial screening are consistently better than their alternative counterparts using routine cancer registry only.

For people having a negative colonoscopy result, a repeated colonoscopy every ten years can produce a more cost-effective result: annual colonoscopy is too expensive. The effectiveness and cost-effectiveness as measured by ICER for repeated colonoscopy at a ten-year interval are consistently better than those with annually repeated colonoscopy under corresponding scenarios (*A_1_ vs A_3_*; *A_2_ vs A_4_*; *B_1_ vs B_3_*; *B_2_ vs B_4_*).

Compliance rates have a significantly impact on the total cost and effectiveness of CRC screening programs. In previous studies, compliance rates for FOBT and colonoscopy were often estimated for modeling [25]–[28]. In this study, we built our models using real observational data. Meanwhile, we tested the impact of compliance rates on the models by varying the rates from 30% to 100%. We found that the compliance rates of our study participants are lower compared with findings undertaken elsewhere in China [29]. Zheng et al [29]achieved 87.4% coverage of FOBT+HRFQ screening and 76.6% compliance for colonoscopy requests in a rural Chinese population, significantly higher than those of this study population. However, under the preferred *Scenario B_1_*, the cost-effectiveness of the screening program would remain virtually unchanged if similar compliance rates were achieved in our study population because the increase of FOBT+HRFQ coverage would lead to a slight decrease of ICER; whereas, a slight increase of ICER would appear when compliance with colonoscopy increases.

It is unclear what contributed to the low compliance rates for CRC screening in our study population: further studies are warranted. Experiences of developed countries demonstrated that to reduce financial barriers and ensure equal access to those cancer screening programs are better financed by governments [30]–[33]. Empirical evidence shows that improved understanding of CRC screening can encourage people to comply with prescribed procedures in screening programs [34], [35]. Unfortunately, CRC screening guidelines freely available to the public in some developed countries remain unavailable in China.

In China, cervical and breast cancer screening programs have been included in public health services for rural populations since 2009and a good cost-effectiveness has been presented[36], [37].Based on evidence support of this study, we suggest that CRC screening be included in the public health services list.

Compared to previous studies, this study has some unique characteristics. It is worthy to note that the combined use of FOBT and HRFQ as initial screening for CRC is an original development in China. To our knowledge, this is the first study of its kind attempting to evaluate the cost-effectiveness of CRC screening programs in urban Chinese populations. The core data used for the simulation modeling came from real observational data.

## Limitations

In this study, we only calculated direct costs. Indirect costs such as those associated with production loss due to attending screening and treatment services should be considered in future studies.

## Conclusion

A combined use of FOBT and HRFQ is preferable in CRC screening programs as an initial screening instrument. Annual FOBT+HRFQ screening is recommended for those who have a negative initial result and those who have a positive result but have failed to comply with colonoscopy procedures. Repeated colonoscopy (for those with a positive result in initial screening but a negative colonoscopy result) should be performed at a ten-year interval instead of one-year.

The current Chinese Trial Version for CRC Screening Strategy falls into *Scenario B_4_*, which is one of the least cost-effective options and should be revised in line with *Scenario B_1_*.

## Supporting Information

Figure S1 **Markov process for CRC screening protocol one (** ***Scenario A_1_–A_4_*** **) using FOBT as initial screening procedure.** Transitions to different Markov states (in oval) are described, with normal, polyp and CRC as transient states and death as an absorbing state (patients cannot leave). The parameters used in the model were described in Table 1. Note: CRC - Colorectal Cancer; FOBT - Faecal Occult Blood Test; NC - No Compliance.
(TIF)

Figure S2 **Markov process for CRC screening protocol two (** ***Scenario B_1_–B_4_*** **) using FOBT+HRFQ as initial screening procedure.** Transitions to different Markov states (in oval) are described, with normal, polyp and CRC as transient states and death as an absorbing state (patients cannot leave). The parameters used in the model were described in Table 1. Note: CRC - Colorectal Cancer; FOBT+HRFQ -Faecal Occult Blood Test + High-Risk Factor Questionnaire; NC - No Compliance.
(TIF)
