# Supplementary figures and images for: Cost-Effectiveness of Colorectal Cancer Screening Protocols in Urban Chinese Populations
Source: PLoS One. 2014 Oct 6;9(10):e109150. doi: 10.1371/journal.pone.0109150 (PMC4186806; doi:10.1371/journal.pone.0109150)

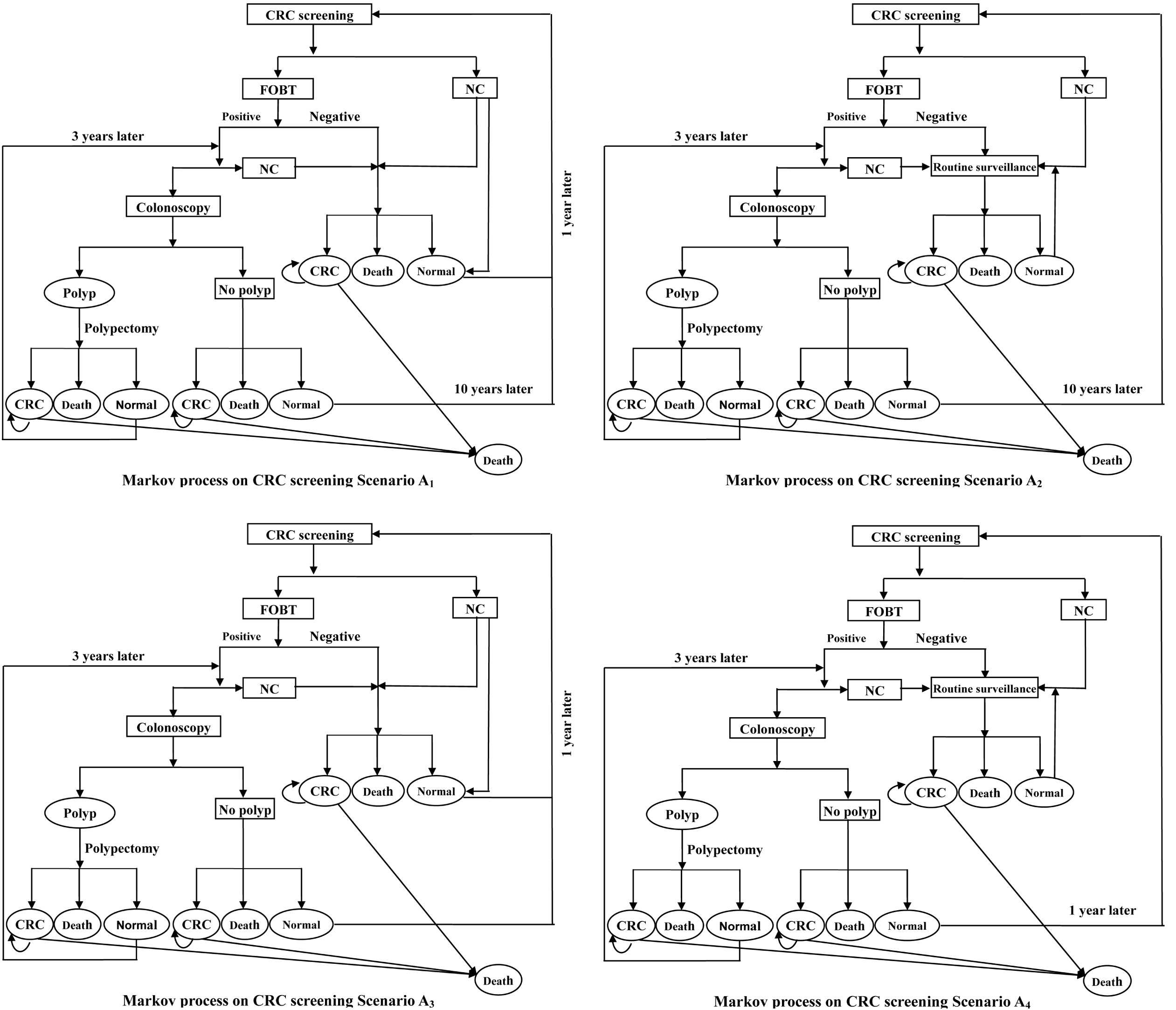

Supplement: Figure S1 — Markov process for CRC screening protocol one ( Scenario A1–A4 ) using FOBT as initial screening procedure. Transitions to different Markov states (in oval) are described, with normal, polyp and CRC as transient states and death as an absorbing state (patients cannot leave). The parameters used in the model were described in Table 1. Note: CRC - Colorectal Cancer; FOBT - Faecal Occult Blood Test; NC - No Compliance. (TIF) [file pone.0109150.s001.tif]

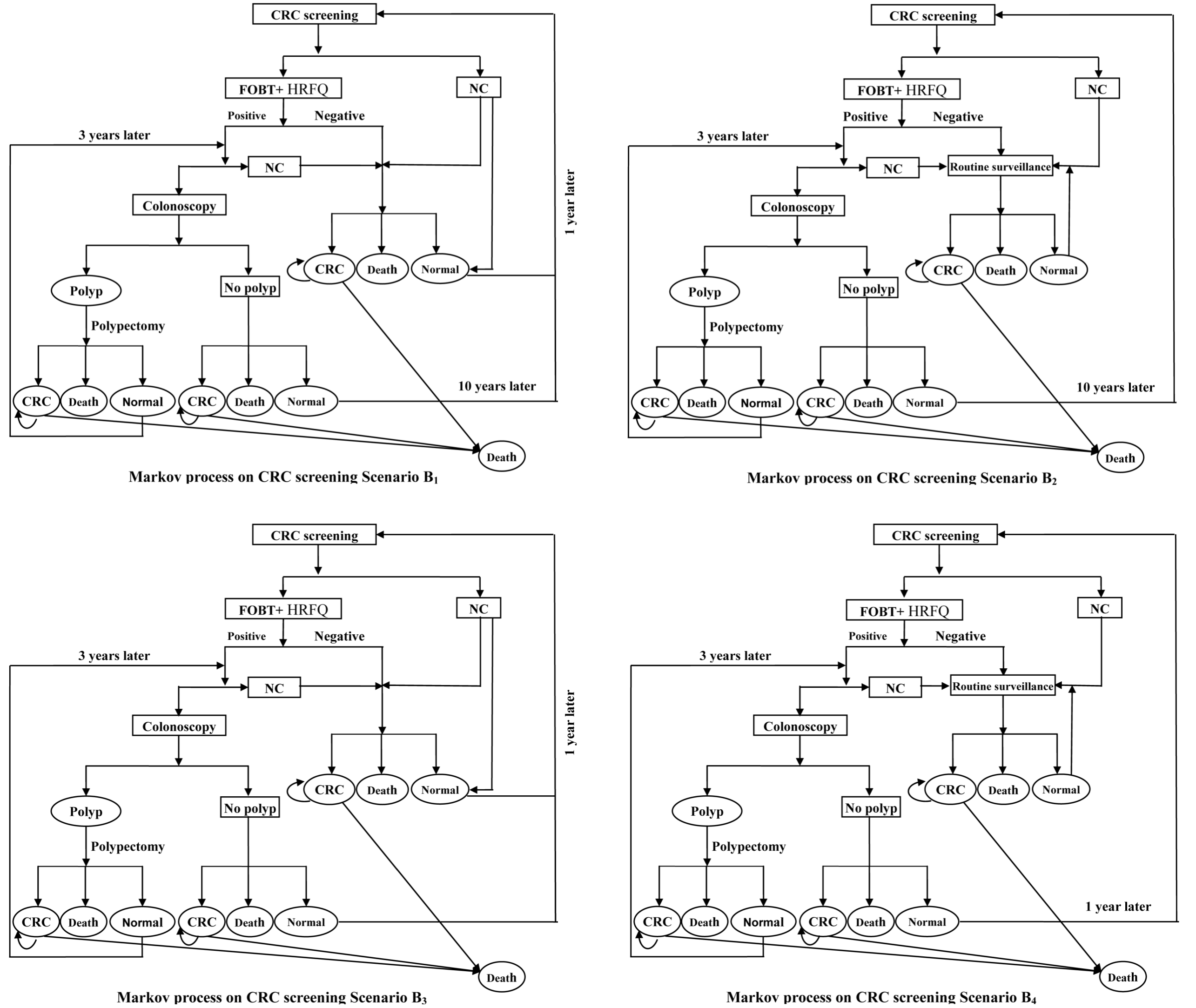

Supplement: Figure S2 — Markov process for CRC screening protocol two ( Scenario B1–B4 ) using FOBT+HRFQ as initial screening procedure. Transitions to different Markov states (in oval) are described, with normal, polyp and CRC as transient states and death as an absorbing state (patients cannot leave). The parameters used in the model were described in Table 1. Note: CRC - Colorectal Cancer; FOBT+HRFQ -Faecal Occult Blood Test + High-Risk Factor Questionnaire; NC - No Compliance. (TIF) [file pone.0109150.s002.tif]
